# A citizens' jury on euthanasia/assisted dying: Does informed deliberation change people's views?

**DOI:** 10.1111/hex.13008

**Published:** 2019-12-09

**Authors:** Simon Walker, Richard Egan, Jessica Young, Chrystal Jaye, Christopher Jackson

**Affiliations:** ^1^ University of Otago Dunedin New Zealand

**Keywords:** assisted dying, citizens' deliberation, citizens' jury, euthanasia, health policy, New Zealand

## Abstract

Euthanasia or assisted dying (EAD) remains a highly contentious issue internationally. Although polls report that a majority New Zealanders support EAD, there are concerns about the framing of the polling questions, and that those responding to the questions do not know enough about the situations described, the options available and the potential implications of EAD policy. One way to address these concerns is through a citizens' jury, which is a method of learning how a group of people view an issue following informed deliberation. This citizens' jury was conducted to learn whether a group of 15 New Zealanders thought the law should be changed to allow some form of EAD and the reasons for their view, having been informed about the issue, heard arguments for and against, and having deliberated together. The jury met for two and a half days. They did not reach a consensus, but become polarized in their positions, with several changing their positions to either strong opposition or strong support. The reasons why people support or oppose EAD were not reducible to particular principles or arguments, but reflected an integrated assessment of a range of considerations, informed by personal priorities and experiences. These results suggest that views on EAD may change in response to informed deliberation that the EAD debate involves a range of value judgments and is not likely to be resolved through deliberation alone. These results may inform international debate on EAD policy.

## 1 INTRODUCTION

Euthanasia or assisted dying (EAD) remains globally one of the most divisive issues in medical ethics and health‐care policy. While the number of jurisdictions allowing some form of EAD has increased in recent years, the debate is far from being resolved. In a democratic society, policy disputes may be settled through a vote, either amongst elected representatives or the general population. However, with complex issues such as EAD, there is a concern that the polling question may be vulnerable to framing effects (ie, that responses may be subject to the wording of the question) and that voters may have an incomplete or inaccurate understanding of the issue.^1^, ^2^ There is also concern that they may not understand the range of other end‐of‐life options currently available or what the possible implications of policy change may be.^1^

In an attempt to address these concerns, other methods of collective decision making have been explored. One such method is a citizens' jury. This is a method of understanding how members of the public view a complex issue, whether their opinion is responsive to informed deliberation, and what reasons they consider significant in forming their views. Internationally, this method is increasingly being used in relation to health policy issues.^3^, ^4^ It involves selecting a group of people to learn about and subsequently deliberate on a particular issue or question. As a form of citizens' deliberation, citizens' juries can enhance democratic processes by enabling informed, respectful debate on social policies.^5^ They have the potential to increase political legitimacy by enabling a greater variety of people to be meaningfully involved in decision making (particularly in areas of social policy where attitudes and values are of central concern) and can encourage a broader understanding of different perspectives. They may, in turn, lead to the resolution of disagreements or clarify what disagreements are based on.^3^

A citizens' jury generally involves non‐experts being brought together to hear arguments and deliberate together.^6^, ^7^ Expert witnesses are selected to provide key information and present different sides of the ‘case’ to the group. An independent steering group representing important stakeholder groups is also appointed to oversee various aspects of the project; this group reviews the information to be provided to the jury to ensure that it is accurate and balanced, and advises on the wording of the overarching question, the selection of experts and the interpretation of the outcome.

Some of the arguments for and against EAD are complex and nuanced, though as Emmanuel showed in a 1994 summary of historic and contemporary debate, the key claims of each side have remained broadly consistent over a long period.^8^ In our reading of the debate since this paper was published, the arguments have followed the same form. The arguments in support of EAD typically appeal to compassion and autonomy. Compassion‐based arguments maintain that EAD is necessary to address unbearable suffering, while arguments based on respect for autonomy hold that people should be permitted to decide whether their lives are worth continuing, and to have lawful access to a safe means of ending their lives if they so choose. Arguments against EAD are more varied, but still centre on certain recurring themes. Some claim, for instance, that it is morally wrong to intentionally kill a person in the circumstances being considered or to assist suicide and that health‐care providers in particular have a duty to sustain the value of human life in suffering. Others focus on the potentially wider implications of the practice, such as the possibility that those who are vulnerable could be pressured into ending their own lives, or that the practice would reduce patients' trust in health‐care professionals, or otherwise reduce the quality of health‐care services. The recurrence of these arguments for and against has been reported in a recent study of social media posts on the debate.^9^

Debate around these arguments is often confounded by a lack of agreement about what key terms and concepts mean and how they should apply, including how the various practices should be described. Here we are using the acronym EAD as the most general descriptor, but acknowledge that the terms represented may not be universally acceptable. We use the term ‘euthanasia’ to mean a lethal injection that is administered at the voluntary request of a competent patient by a doctor or a nurse practitioner. ‘Assisted dying’ refers to a doctor providing a prescription for lethal medicine at the voluntary request of a competent patient, and the patient then self‐administering the prescription at the time of their choosing. Another confounding factor is the breadth of practices and situations to which the debate may apply, and disagreement about how they compare. There is, for instance, on‐going disagreement about how actively killing a person differs from withdrawing or withholding potentially life‐extending treatment. There is also disagreement about what kinds of suffering justify EAD, and whether consent could be given via an advanced directive. If a form of EAD is permitted, the relevant policies must specify under what conditions it should be available and what safeguards should be in place to ensure that these conditions are properly met. It must also specify who can provide it and how, and what processes should be in place to ensure public safety. All these considerations make the debate particularly complex and challenging to resolve.

In New Zealand, it is currently illegal for a doctor to intentionally bring about a patient's death under any circumstances or to assist a person in ending their own life. However, public awareness of and debate on these issues have intensified in recent years.^10^ In 2017, a Parliamentary Health Select Committee reported on a two‐year investigation of public attitudes on the issue, which considered over 21 000 submissions. The report showed both strong support for and opposition to a law change, and that the concerns and arguments raised by submitters closely correspond to those raised in international debate.^11^ In June of the same year, a Bill to change the law to make EAD lawful was drawn from the Members' Bills ballot.^12^ This Bill passed its first reading in December 2017 and was referred to the Justice Select Committee. This Committee received over 39 159 submissions.^13^

We conducted a citizens' jury in March of 2018 to learn whether a group of New Zealanders would support or oppose a change in the law to allow EAD, and the reasons they considered important in forming their views, having received information about the issue, considered arguments both for and against it and having deliberated amongst themselves. The purpose of this inquiry was to understand more clearly why people disagree about EAD, to learn whether their views would change through informed deliberation and to use this knowledge to contribute to the Select Committee process and international debate.

## 2 METHOD

### 2.1 Steering group

The steering group for this citizens' jury included people with different views on whether the law should be changed to permit EAD. It consisted of a palliative care specialist, a legal expert, a medical ethicist with specialist knowledge of EAD and a former Health and Disability Commissioner. In addition, each member has significant societal credibility and respect at a national level. The wording of the question for the jury was carefully considered by the steering group so as to achieve accuracy and avoid prejudice. The question was whether the law in New Zealand should be changed to allow doctors to provide or administer a medicine to a person, at their voluntary and competent request, that will bring about their death, under certain circumstances. The jury was also asked to specify the main reasons for its answer, the conditions under which the actions should be legal if it supported a law change, possible exceptions to prosecution if it opposed a law change or the main points of disagreement if it could not agree.

### 2.2 Experts

Seven experts were chosen to represent seven key aspects of the debate: the current law in New Zealand regarding end‐of‐life decisions, the nature of palliative care and the palliative care perspective on EAD, the main ethical arguments for a law change, the main ethical arguments against a law change, a disability perspective, the perspective of a family member of someone who recently died and publicly sought law change around EAD and a Māori perspective (indigenous New Zealander). The experts were each asked to speak for 20‐30 minutes and to answer questions from the jury. They were given general guidance on what to include in their presentations, but the specific content was left to their judgment. Where appropriate, the experts were asked to present the majority view of the constituencies they represented and to signal where they held personal views or interpretations, one way or another, on the particular aspect of the EAD debate about which they were speaking.

An independent chair was appointed to manage the hearing. A facilitator was engaged to ensure each member of the jury was able to participate and to prevent the discussion from being dominated by an individual or group. The chair was an academic experienced in managing meetings, and the facilitator was an expert mediator employed by the University of Otago.

### 2.3 Recruitment

A random sample of 151 people from the study area's electoral roll and from the Māori electoral roll were sent letters of invitation to participate. The letters explained the nature of the process and what would be required of the jurors, including the need to be willing to discuss the issue in an open and respectful manner. We chose a random selection method to mitigate selection bias. Those who identified as having strong fixed positions were asked not to participate as there was concern such views might inhibit open discussion. For the same reason, we decided not to include people who might be viewed as ‘experts’ by other jurors (such as doctors). These characteristics were screened for in the participation forms. We attempted to follow‐up invitees by phone. Two hundred dollars of supermarket vouchers were offered to each participant to cover expenses such as travel and childcare, and in recognition of the substantial time commitment involved.

### 2.4 Jury process

The jury took place over four days at a neutral public venue and included two half days and one full day meeting. It has been suggested that four to 5 days is necessary to allow sufficient time for deliberation.^3^ It was decided that a shorter time was necessary to encourage a range of participants, given the relatively small amount of money available for reimbursement. The meetings were not over consecutive days as it was decided that a break would help jurors to consider what they had heard and their positions. The group first met on a Wednesday afternoon. At this meeting, the chair introduced the research team and the facilitator, and a member of the research team gave an overview of the process and the aims of a citizens' jury and introduced the question. The jurors were asked to try to consider the question from the standpoint of ordinary New Zealand citizens, that is as members of a secular, pluralist democracy, where respect for differences and compromise are requirements of public life. At the same time, they were encouraged to discuss the issues in a robust, respectful and rigorous manner, to state their views honestly and to ask difficult questions of the experts and of each other. The jury then listened to the medical law specialist speak about the current law in New Zealand regarding end‐of‐life decisions. Some members of the jury asked questions about the End of Life Choice Bill being considered by parliament and how it might be interpreted. This expert took a neutral stance on the issue and did not attempt to persuade the jury as to how they should decide. The expert representing palliative care spoke next about current practice, including the goals of palliative care and what is currently achievable in symptom management. This expert explained why most in the hospice and palliative care community are opposed to EAD, but added that, if the law were to be changed, she would personally favour a system similar to that established in Oregon, where doctors are not actively involved in the administration of lethal medication. Each speaker was available for 15‐30 minutes after their presentations to respond to questions from the jury, and the jury had 30 minutes at the end of the day to speak together with the facilitator present.

The jury did not meet the following day, but were encouraged to carefully consider what they had heard and discussed so far, and to prepare questions for the next meeting. On Friday of that same week, the jury heard from the remaining five experts. The main ethical arguments for and against EAD were presented in the morning; these covered familiar themes as seen in international debate, along with the key issues raised through the Health Select Committee process.^11^ The three experts representing particular perspectives presented in the afternoon. The speaker from the disabled community spoke against a law change, primarily on the basis that the conditions under which EAD is generally considered justified are often applicable to people with disabilities, and he believed promoting this option would undermine individual and community efforts to affirm and sustain the value of life with a disability. The speaker providing a Māori perspective also spoke against a law change. He believed that EAD is highly problematic to the Māori understanding of death because it interrupts the dying process. He described the social, economic and health disparities experienced by Māori and argued that some people may be driven to choose EAD because of these disparities and that this would be unjust. The family member spoke in favour of a law change on the basis of the suffering her loved one endured at the end of her life and of the lucidity of that loved one's desire to have the option of EAD. All of the speakers on this day conveyed their personal feelings with regard to this issue, while expressing themselves in a reasonable and respectful manner.

The jury met again on Saturday morning at 9:30 to deliberate, with assistance from the facilitator. The research team was not present for the deliberations, but was called in by the jury at midday to receive the verdict. At the time, the facilitator summarized the key points of the deliberations.

### 2.5 Questionnaires

The jury members individually completed two questionnaires, one at the beginning of the first meeting and the other after having reported the verdict. The first questionnaire collected demographic data, including age, gender, ethnicity, religion (including attendance), level of education and employment. Both questionnaires included the following Likert‐scale question, taken from the New Zealand Attitudes and Values Study^14^ (which is derived from the British Attitudes and Values Survey): ‘Suppose a person has a painful incurable disease. Do you think that doctors should be allowed by law to end the patient's life if the patient requests it?’ The possible responses range from 1 (definitely NO) to 7 (definitely YES). The post‐jury questionnaire asked jurors to answer this question again and, if their answer had changed in the time since the process had begun, to provide reasons for this. It also included a question about their experience of the jury process. Both questionnaires also asked whether the issue should be addressed through a national referendum (as had been proposed by one political party in New Zealand).

### 2.6 Ethics approval

Category B (departmental level) ethics approval was granted (D18/076) and then audited and approved by the University of Otago Human Ethics Committee.

### 2.7 Reporting

After the jury, the steering group reconvened with the research team to discuss the results and advise on how they should be reported. The research team then produced a report of the process and outcome as a submission to the Justice Select Committee considering the End of Life Choice Bill. The jury members were emailed a copy of this report before it was submitted, and were invited to provide feedback as to whether they agreed with how it described the process and outcome.

## 3 RESULTS

### 3.1 Participants

Seventeen of the one hundred and fifty people invited to participate indicated that they were willing to take part; of these, 15 were selected. This selection aimed at bringing the group more in line with the sample frame, by selecting a man over a woman and a younger person over an older person. Ninety‐three people declined to participate, with most citing practical reasons such as work or family commitments. One declined because they did not believe they could discuss the topic in a sufficiently open‐minded way, as their feelings about it were too strong. The remaining forty could not be contacted.

All 15 jurors remained for the whole process. There were four men and eleven women on the jury, amongst whom ages varied: two were between the ages of 18‐29, ten between the ages of 50‐69, and three were aged 70+. Fourteen jurors identified as NZ European and one as Māori. Religions identified amongst the jury included Buddhist (1), Christian (6) and ‘none’ (8). Eleven stated they never attend church, three said annually and one ‘when possible’. Employment included retired, stock agent, lecturer, self‐employed, nursing, personal assistant, retail, caregiver, council, coordinator, personal trainer and contractor. Qualifications were reported as follows: no qualification (3), high school qualification (3), certificate (3), diplomas (3), bachelor's degree (1) and postgraduate degree (2). This group is older than the general population, has proportionately more females (73% compared to 51%), more New Zealand Europeans (93% compared to 74%) and slightly more identifying as having no religion (53% compared to 47% amongst New Zealand Europeans in the general population).^15^

### 3.2 Deliberation

**Figure 1:**
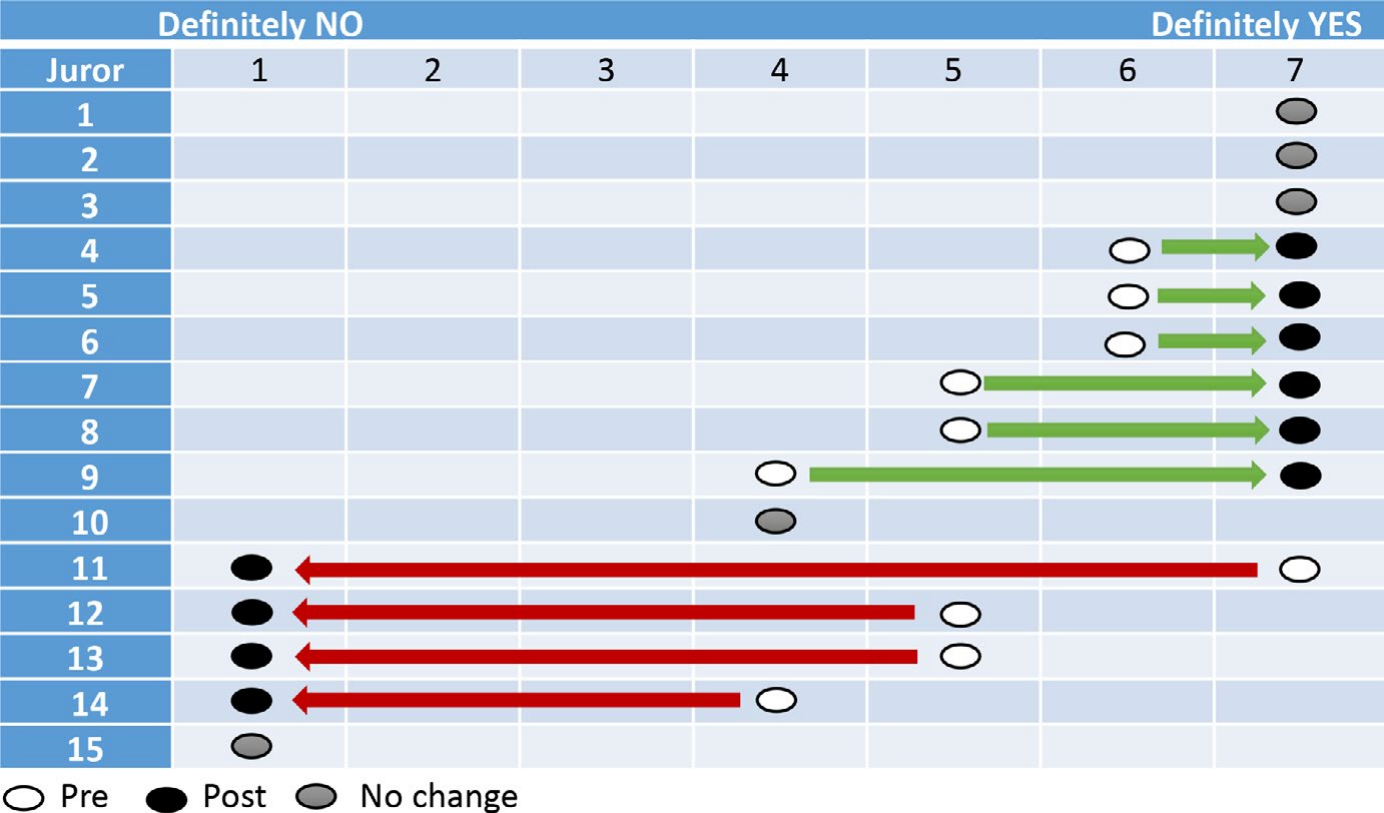
The jurors’ views as reported in the pre‐ and post‐jury questionnaire. Question: ‘Suppose a person has a painful incurable disease. Do you think that doctors should be allowed by law to end the patient's life if the patient requests it?’

At the end of their deliberations on the Saturday morning, the jury reported that they could not reach agreement about whether the law should be changed. This is an unusual outcome for a citizens’ jury. The deliberative process ordinarily moves participants towards agreement, but in this study it appears to have strengthened the disagreement.^3^ The first questionnaire, completed prior to the presentations delivered by experts, had indicated that a majority were in favour of a law change, while some were uncertain and one member was opposed. The second questionnaire, which was completed following the verdict, showed that nine jury members were firmly supportive of a law change, while five were firmly opposed. One indicated uncertainty on the second questionnaire, but stated that it would depend on the conditions under which EAD would be available, and stipulated that they would only support EAD for situations where the person had a terminal condition. Of the nine who were firmly supportive, six had moved from positions of uncertainty or moderate support. Of the five who were opposed, four had moved from positions of uncertainty or support. These changes are illustrated in Figure 1. In the pre‐jury questionnaire, twelve jurors stated that the issue should be addressed by a general referendum. In the post‐jury questionnaire, this number was reduced to nine.

When reporting back to the research team after the deliberations on Saturday, those in favour of a law change and those opposed cited several reasons for their respective positions. These reasons for and against had been noted on a whiteboard by the facilitator, and these notes were used to aid jurors in their recollection of their deliberations (see Table 1). Those supporting a law change recognized several of the concerns raised by those who were opposed, but believed that these could be adequately addressed through safeguards, for example by engaging specialist mental health practitioners to assess the voluntariness of a decision. They also maintained that allowing EAD would not preclude increasing funding to the health system and that it is unfair to expect those who need EAD now to wait for the health system to be improved.

**Table 1:** Jurors’ reasons for and against a law change, as listed on whiteboard at end of the deliberations

What are your main reasons for supporting a law change?	What are your main reasons for NOT supporting a law change?
Emotive plea from expert To prevent people having to endure unbearable pain Maintain quality of life Respecting individual choice and beliefs Dignified death on own terms Protecting loved ones from witnessing suffering and allow family comfort and preparation for death Clarity for doctors and lift burden of existing grey area around hastening death Compassion Putting the patient at the centre of decision making Knowing the option is available—comfort of choice Health system does not fund adequate palliative care for everyone Currently: freedom from decision—instead—need freedom to decide	Concern that parliament will not have considered all possibilities arising out of a law change Law once passed difficult to go back o for example, funding would influence decisions about overturning legislation. Once law passed resources will be channelled into euthanasia/assisted dying and less given to palliative care. Any restrictions and safeguards in the legislation would be eroded over time due to legal interpretation Elder abuse Coercion and pressure by others Sufficient care currently exists for a good death System is not broken but do need more funding for palliative care Concern that it would be too easy to meet the criteria to end life Vulnerable (elderly, disabled etc) made to feel worthless and society more accepting of that Fail to recognize a vulnerable state of mind Concern insufficient counselling around end‐of‐life decision making and how it would feel for the individual to make that decision. What is it like for the family left behind Can a painless death be assured Law change is only for a minority but impacts on a majority Ethical issues for the medical profession

Several of the reasons just described were reiterated in the post‐jury questionnaire by jurors explaining why their views had changed, though some of these comments were more personal and cannot be reduced to a single reason (see Box 1). In describing their experiences of the jury process, jurors were almost entirely positive and several expressed gratitude for the opportunity to participate, to become more informed on the issue and to be able to contribute to the debate. This positive assessment of the process aligns with the observations of the facilitator and the members of the research team who sat in on expert presentations, who reported that the jurors remained receptive to the information presented and asked a number of questions of the experts and the research team. The facilitator also reported that in their deliberations the jurors conveyed deep concern over the issues that were raised, while remaining respectful of competing viewpoints.

Box 1 **Reasons provided in post‐jury questionnaire for why jurors changed their views**

**Table:** 

‘Once the emotive reasons were excluded my life teaching took over’ (Participant 1) ‘I believe that there is enough care in NZ for people for this bill not to go forward. It will end up being abused and if it is introduced, you can never go back’ (Participant 8) ‘The ability to include safeguards and conditions. Key speakers provided useful information’ (Participant 10) ‘This has changed because of all the areas we discussed for and against. There seemed too many grey areas, eg family not involved in decision, is it actually a painful death? That it may make it too easy for people, what was their state of mind? How you would actually feel yourself about making the decision to go through with euthanasia’ (Participant 11) ‘After considering safeguards for the proposed new law the negatives did not outweigh the positives. The negative points were trivial compared to the positive effects’ (Participant 12) Always supported, no change (Participant 13) ‘first view from sick person's point of view impact of change law too great on society as a whole. Don't trust society to not water down restrictions put in place’ (Participant 14) ‘After evaluating all the facts, I realized that in the end I would prefer the choice to be able to be made by the patient and that's why I want to legalize it’ (Participant 15)

### 3.3 Review and reporting

A written report of these results was prepared with advice from the steering group and was submitted to the Justice Select Committee considering the End of Life Choice Bill. Members of the research team also spoke to the Committee in person about the study. None of the jury members asked for changes to this report when given the opportunity, and a small number positively confirmed that it faithfully represented the process and outcome.

## 4 DISCUSSION

A group of 15 New Zealanders served on this citizens' jury on EAD, deliberating together about whether the law should be changed in NZ to allow EAD. They were provided with detailed and accurate information about the issue and listened to experts present arguments from both sides of the debate. Informed deliberation did not bring them to a unanimous decision, but instead made them firmer and more polarized in their views. A number of the jurors changed from being either uncertain or supportive, to being strongly opposed, while others moved from being uncertain or moderately supportive to being strongly supportive. These results suggest that views on EAD may change in response to informed deliberation. They also suggest that the public debate will not be resolved through this process and that there is not a position that will be acceptable to all sides.

A possible limitation of this study is that the jury process was constrained to a four‐day period. This may not have provided the jury with sufficient time to process the information they were given, and some of the jurors did indicate that they would have benefited from more time to deliberate. Some citizens’ deliberation projects involve a week or more between sessions, so as to allow for more extended personal deliberation.^3^ This project was held over a shorter period to increase the chances of gathering a sufficient number of participants, given the relatively low amount of remuneration that was offered. Moreover, while it is possible that with more time to deliberate the jurors might have further changed their views or reached consensus, the polarization of views shown by participants’ responses to the post‐jury questionnaire suggests that this was unlikely, as it indicates that their views were becoming more entrenched. In support of this, the facilitator observed that the participants had become settled in their views by the end.

Raisio and Vartiainen have discussed the intractability of the EAD debate as an instance of what social policy theorists Rittel and Webber have termed ‘wicked problems’.^16^, ^17^ They maintain that such problems are ‘well‐nigh impossible’ to solve in a lasting and generally acceptable way due to the combination of difficulties they present, including disagreements about formulating the question, an inability to test possible answers, connections to other unresolved problems, the lack of objective criteria against which to assess claims and the range of competing values that shape how people view and respond to them. This characterization might explain the outcome of this citizens’ jury, in that it indicates the difficulty of reaching agreement on such an issue through deliberative processes. However, other citizens’ juries have reached agreement on complex moral problems, which suggests that the complexity alone does not account for the disagreement.

A key difference between this citizens’ jury and others where agreement on complex problems has been found may be in how the jury's task was presented. The purpose of this study was to understand why people disagree about EAD and whether agreement could be achieved through informed deliberation. Though the jury was asked to attempt to work towards an agreed position, disagreement was presented as a possible outcome. An alternative approach could have been to ask the jury to focus on finding a compromise position, after disagreements had been identified. In other words, the task of the jury could have been to identify a position that conflicting parties could ‘live with’, given their sustained disagreement. This was not the primary goal of the project and would likely have required more time, as the jurors would have first needed to consider what their ‘informed’ positions were (having heard from the experts and deliberated) and to have then deliberated on a compromise.

The degree of emphasis given to reaching a consensus has been identified as a ‘crucial decision in the design and facilitation' of a deliberation process.^18^ This decision relates to an apparent tension in deliberative methodology between openly investigating different standpoints and generating an agreed position, which may necessarily exclude certain positions, in the sense that agreeing to compromise requires letting go of what one may view as an ideal position.^19^ This in turn may be related to the problem of distinguishing political and moral questions. In this citizens’ jury, the jury were charged with examining the question from the standpoint of ‘New Zealand citizens’, and this was explained as meaning (amongst other things) that the jurors should try to base their position on values that are common to New Zealanders, and not solely on personal convictions, such as religious beliefs. Accordingly, in their questions and reporting the jurors did not raise religious‐based arguments. However, while this may be because the jurors were attempting to follow the instructions, it could also be because religious observance was lower amongst this group (though the proportion of jurors who identified as Christian is similar to that of the general population, those jurors who identified as such reported minimal church attendance).^20^ Moreover, despite the instructions, it is questionable how far one can set aside ‘personal’ convictions in considering an issue such as this, as how a person assesses given arguments often depends on prior moral judgments.

The problem just indicated can be illustrated in the differing ways jurors assessed the purported risks of EAD. In their deliberation, all of the jurors agreed on the need to take potential risks into account, and yet they disagreed about whether these risks could be safely managed, and how they should be weighed against the needs of those who would benefit from a law change. In certain respects, this could be viewed as a disagreement about facts, that is, facts about how many people would actually be at risk (if any), compared to the number of people who could benefit. It is possible to look for such facts by studying trends and outcomes in those countries that have an EAD policy in place.^21^ However, while this may settle certain disagreements, how a person interprets such facts will depend on prior judgments about the kinds of cases for which EAD is acceptable and how purported risks and benefits should be weighed.^22^, ^23^

A common approach to explaining moral decisions is to base individual judgments on general moral concepts, such as autonomy or compassion. On this model, the normative force of a given argument depends on its grounding in general principles. However, some philosophers argue that the normative force of such concepts is dependent on a background of commitments grounded in practices.^24^ On this view, a moral principle is not a justificatory bedrock but a way of describing a general or shared value, which is inherent in the form of life that a person inhabits. An implication of this view is that it is often impossible to reduce a person's adherence to one or other moral position to a single principle. This seems to be reflected in the range of reasons the jurors put forward in explaining their respective positions, and in the written explanations offered by jurors who had changed their views in the post‐jury questionnaire (Box 1). In those written comments, several cited a cluster of arguments or referred to a kind of holistic and personal assessment, in terms of, for example, how far they ‘trust society’, the ‘realization’ of what they would prefer or ‘life teaching taking over’. This suggests that the reasons why people support or oppose EAD are not reducible to particular principles or arguments, but reflect an integrated assessment of a range of considerations, informed by personal priorities and experiences.

When informed deliberation fails to produce agreement, and further information is not likely to resolve the impasse, it may be that the only reasonable response left in a democratic society is to revert to a vote.^5^ Though there was an increase in the number of jurors opposed to a law change after the deliberation process, there was still a majority in support. Similarly, though fewer jurors supported a general referendum on the question after the deliberation, a majority still supported this as a way of settling the issue. To insist on unanimous agreement before changing, policy could be to prejudice policy in favour of the status quo.^5^ However, a number of the jurors in this study modified their position through the deliberative process. This indicates the need to ensure that voters are adequately informed before voting, but given the complex nature of the topic, this would be very difficult to achieve. This is a reason for not addressing the issue through popular referendum, but for relying instead of informed, elected representatives.

Though we sought to mitigate selection bias by randomly selecting potential participants from the electoral role, we think a degree of self‐selection bias is unavoidable in research of this nature. The jurors' responses to the New Zealand Attitudes and Values Survey question at the beginning of the process were close to what has been found amongst the general population: the national survey reports that 66% of respondents are supportive, 21.2% are unsure, and 12.3% are opposed.^14^ The reasons they gave for their respective views correspond closely with arguments laid out in the NZ and international literature.^10^, ^21^

The available survey data indicate that there are no statistically significant demographic associations with attitudes to EAD amongst New Zealanders relating to age, gender or economic status, but associations with religiosity, educational attainment and certain ethnic groups.^10^ Given these findings, and given the concerns regarding the reliability of surveys in gauging views on this matter, it is possible that certain groups would respond differently to the perspectives and arguments put forward by the experts and that their deliberation would produce a different result. This could be tested by repeating the process with selected groups.

## 5 CONCLUSION

A citizens' jury was conducted to examine whether a group of New Zealanders believed the law should be changed to allow some form of EAD, having been presented with relevant information, arguments and perspectives, and having deliberated together. Rather than reaching a consensus, the group became polarized in their views and cited a range of reasons for their respective positions. This finding suggests that the EAD debate involves a range of complex and personal value judgments and is not resolvable through rational deliberation alone. The deliberative process remains useful as a means of improving understanding, conveying the nature of the disagreement and clarifying the decision that needs to be made. Further research into what the contrasting value judgments are based on, and whether they differ between groups, would be useful. The finding also suggests that a vote may be the only reasonable way of deciding the issue. However, that several of the jurors changed their views through the deliberative process emphasizes the need for voters to have an adequate understanding of the issue before voting, and the practical difficulty of achieving this is reason to entrust the vote to elected representatives.

## CONFLICT OF INTEREST

No member of the research team has a conflict of interest in regard to this research.

## Data Availability

The qualitative data that support the findings of this study are not publicly available due to privacy or ethical restrictions. A copy of the pre‐ and post‐jury survey data is available on request from the corresponding author. The information booklet provided to the jury, which includes a list of the expert witnesses, is available at http://hdl.handle.net/10523/9382.
